# An investigation into the range dependence of target delineation strategies for stereotactic lung radiotherapy

**DOI:** 10.1186/s13014-017-0907-8

**Published:** 2017-11-03

**Authors:** Dennis J. Mohatt, John M. Keim, Mathew C. Greene, Ami Patel-Yadav, Jorge A. Gomez, Harish K. Malhotra

**Affiliations:** 1 Department of Physiology and Biophysics, University at Buffalo, NY, Buffalo, 14214-3005 USA; 2 Department of Radiation Medicine, Roswell Park Cancer Institute, NY, Buffalo, 14293 USA

**Keywords:** Stereotactic body radiotherapy, Four dimensional computed tomography, Average intensity projection, Maximum intensity projection, Dice similarity coefficient

## Abstract

**Background:**

The “gold standard” approach for defining an internal target volume (ITV) is using 10 gross tumor volume (GTV) phases delineated over the course of one respiratory cycle. However, different sites have adopted several alternative techniques which compress all temporal information into one CT image set to optimize work flow efficiency. The purpose of this study is to evaluate alternative target segmentation strategies with respect to the 10 phase gold standard.

**Methods:**

A Quasar respiratory motion phantom was employed to simulate lung tumor movement. Utilizing 4DCT imaging, a gold standard ITV was created by merging 10 GTV time resolved image sets. Four alternative planed ITV’s were compared using free breathing (FB), average intensity projection (AIP), maximum image projection (MIP), and an augmented FB (FB-Aug) set where the ITV included structures from FB plus max-inhale/exhale image sets. Statistical analysis was performed using the Dice similarity coefficient (DSC). Seventeen patients previously treated for lung SBRT were also included in this retroactive study.

**Results:**

PTV’s derived from the FB image set are the least comparable with the 10 phase benchmark (DSC = 0.740-0.408). For phantom target motion greater than 1 cm, FB and AIP ITV delineation exceeded the 10 phase benchmark by 2% or greater, whereas MIP target segmentation was found to be consistently within 2% agreement with the gold standard (DSC > 0.878). Clinically, however, the FB-Aug method proved to be most favorable for tumor movement up to 2 cm (DSC = 0.881 ± 0.056).

**Conclusion:**

Our results indicate the range of tumor motion dictates the accuracy of the defined PTV with respect to the gold standard. When considering delineation efficiency relative to the 10 phase benchmark, the FB-Aug technique presents a potentially proficient and viable clinical alternative. Among various techniques used for image segmentation, a judicious balance between accuracy and efficiency is inherently required to account for tumor trajectory, range and rate of mobility.

## Background

The ablative dose involved with stereotactic body radiotherapy (SBRT) requires tumor delineation and dose delivery with the highest level of precision and accuracy [1]. The inter- and intra-fraction motion associated with lung tumors adds an additional level of complexity to the implementation of SBRT in a clinic, for which motion management methods such as deep breath hold, abdominal compression, and respiratory gating have been implemented [2]. However, regardless of method chosen, the final dose delivery to the tumor volume must remain the same to ensure predictable clinical results. Consistent tumor delineation yielding identical planned treatment volumes (PTVs) is the starting point for accurate dose delivery.

Contemporary treatment planning takes into consideration a composite internal target volume (ITV) structure which is defined by the union of individual target volumes at different instances in time. In 2004, Allen et al. described an estimated composite target volume on the basis of the inhale and exhale CT image sets [3]. It is noted in some clinics an “augmented” ITV has further been refined to include the union targets delineated on the free breathing (FB) image set along with the inhale and exhale CT images. Rietzel et al. took this ideology one step further by including the contours of the GTV’s from all 10 phased data sets [4]. This straightforward fusion of GTVs at all instances over the course of respiratory cycle to generate the ITV has since become the “gold standard” approach in the treatment planning process. However, 10 phase target delineation is known to be time consuming when considering alternative methods have been explored which compress the temporal information obtained from 4DCT into a 3DCT data set. The fundamental objective of this study is to determine if efficient ITV delineation strategies are a valid representation for the irradiated target volume.

Alternative methods for ITV generation previously reported include direct segmentation from helical, maximum intensity projection (MIP), or from average intensity projection (AIP) CT image sets [5–9]. In 2006, Bradely et al. compared helical, MIP, and AIP for the purpose of determining the optimal CT based volume methods by looking at the change in spatial isocenter coordinates [5]. Zamora et al. in 2010 quantified the differences of MIP image sets acquired by using various means of phase binning [6]. Han et al. also compared helical and AIP tissue contouring for dose calculation and found differences in mean dose to organs at risk (OARs) within ±5% [7]. In 2011, Speight et al. evaluated a deformable registration segmentation technique to be used in ITV generation for lung patients, saving clinicians a significant amount of time [8]. Tian et al. reported in 2012 on dosimetric comparisons of treatment plans based on free breathing (FB), MIP and AIP CTs [9]. In retrospect, Bradely and Zamora both found ITVs generated via MIP adequate to ensure maximum inclusive tumor extent, whereas Tian and Han recommended AIP datasets be used when target alignment is performed in the presence of asymmetrical respiratory motion, becoming even more problematic when range of target displacements exceeds >1 cm.

To date, only a limited number of studies have compared the differences between CT datasets for lung SBRT treatment planning [4, 6, 8]. Furthermore, few studies have directly compared alternative ITV delineation methods to the gold standard technique, whereas no other study to date has made this evaluation as a function of target range. The purpose of this study is to investigate tumor volume suitability by using manually delineated 10 phased ITV structure as the standard benchmark to compare alternative time saving techniques for lung tumor movement up to 4 cm.

## Materials and methods

**Fig. 1:**
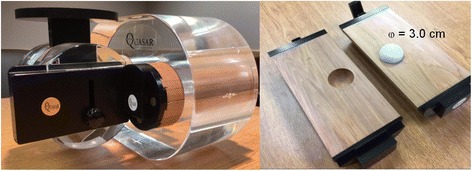
Quasar respiratory motion phantom with cedar lung insert and offset polystyrene sphere 3.0 cm in diameter

In clinical free breathing scenarios, thoracic tumor movement up to 5 cm has been reported with typical cycle rates on the order of 4 s [2]. To model lung-tumor motion we used a Quasar Respiratory Phantom (Modus, London, Ontario, CA) as shown in Fig. 1. The phantom employed a cedar lung insert encapsulating an offset spherical polystyrene target with radius of 1.5 cm. The phantom provided sinusoidal 2D motion along the superior-inferior axis at a programed rate of 15 cycles per minute (4 s respiratory period). The amplitude of target motion was adjusted for ±0.5, ±1.0, ±1.5 and ±2.0 cm translational increments.

### 4DCT image acquisition

**Fig. 2:**
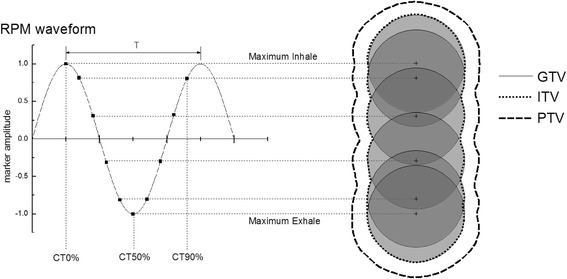
Schematic illustrating the correlation between the phantom waveform with respect to the relative displacement of the GTV target structure

The 4DCT image sets in this study were captured using a GE Lightspeed Pro 16 slice scanner (General Electric, Milwaukee, WI), with a slice width setting of 1.25 mm. At each couch position, projection data was acquired over the duration of the tube rotation plus the additional time to complete at least one respiratory cycle. Respiratory cycle data was subsequently recorded using a Real-time Position Management (RPM) system (Varian, Palo Alto, CA). Briefly, internal target motion was synchronized to external abdominal movement by capturing a reflected infrared illumination signal from a marker block to a CCD camera. The diagram shown in Fig. 2 illustrates the waveform association with GTV movement. The marker trajectory was recorded in real time by RPM software which calculates the respiratory period based on the peaks of the observed amplitudes. Reconstructed images and respiratory data were then transferred to Advantage 4D workstation (General Electric, Milwaukee, WI) where they were sorted and binned with respect to couch position and corresponding respiratory phase at approximately ten uniformly spaced intervals within the respiratory cycle, with CT_0%_ corresponding to the max-inhalation phase and CT_50%_ the max exhalation phase. From these bins, MIP and AIP image sets were also generated by selecting the maximum and average pixel densities across all respiratory phases of the 4DCT data set, respectively. Another helical image was also taken immediately and was referred to as the corresponding free-breathing (FB) scan. The process was repeated by varying the amplitude of the tumor motion to ±0.5, ±1.0, ±1.5 and ±2.0 cm, respectively.

### Target delineation

Manual contouring of the individual GTVs was performed on CT image sets within multiple respiratory phases in order to infer the motion information using Eclipse (version 13.6) treatment planning system (TPS). To avoid any interplanner differences, all contours were segmented by one individual using the same lung window setting in all image sets. Although we are aware of the new automated feature in version 13.6 to create an ITV via accumulations of structures in 4D, the process itself would entail naming each GTV by a common identifier which circumvents discrete phase distinction when preforming DSC analysis. Hence, the benchmark ITV structure was then generated using Boolean “OR” operation to union all 10 GTV phased structures from individual CT image sets corresponding to set motion amplitude. Alternative ITVs were also created by using a single contour outlining the FB, AIP and MIP volumetric image sets. Additionally, in consideration to clinical situations for which 4DCT is not available, an augmented FB (FB-Aug) ITV was generated containing volumetric information from the FB image set fused with the GTV contours at the maximum inhale (CT_0%_) and maximum exhale (CT_50%_) image sets.

**Fig. 3:**
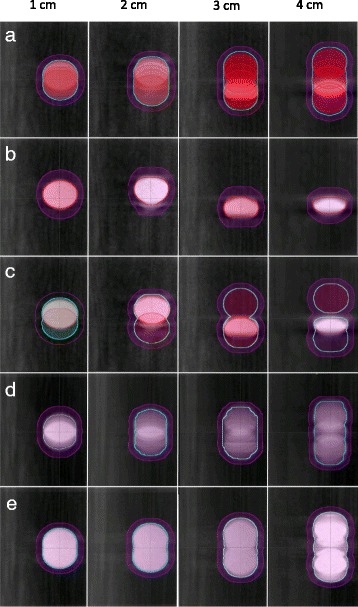
Phantom tumor movement along the superior-inferior direction as viewed in the frontal plane. The red, cyan and magenta contours represent the GTV, ITV, and PTV, respectively. For target ranges from 1 to 4 cm (left to right), the PTV is derived via (**a**) 10 phase “gold standard,” (**b**) free breathing, (**c**) augmented FB, (**d**) average intensity projection, and (**e**) maximum intensity projection image sets

Planned treatment volumes (PTVs) were created in accordance to Radiation Therapy Oncology Group (RTOG) 0915 protocol [10]. In this study we will be comparing the gold standard PTV to alternative PTV’s derived from four methods which include: (1) FB, (2) FB-Aug, (3) AIP, and (4) MIP. In the first scenario, considering only the FB (helical) image set was used, we followed conventional (non-4DCT) protocol by expanding the GTV by a margin of 5 mm in the axial plane and 10 mm along the superior-inferior direction to generate the PTV. Scenarios (2)-(4) followed 4DCT protocol where the PTVs were generated by expanding a uniformly isotropic 5 mm margin from the ITV. Figure 3 illustrates the various PTV’s as derived from the 10 phase, FB, FB-Aug, AIP and MIP techniques. Measurement tools in Eclipse TPS were used to record the volume of the PTV structures.

### PTV ratio analysis

Letting x represent the following methods of target delineation: (1) FB, (2) FB-Aug, (3) AIP, and (4) MIP, a generic PTV ratio can be expressed as:

$$ {R}_x=\frac{PTV_x}{PTV_{GS}} $$

where PTV_GS_ is defined as the “gold standard” PTV generated via fusion of 10 phase gross tumor delineation expanded by an isotropic 5 mm margin.

### Statistical methods

In this study, we used Dice analysis because of its simplicity to preform volumetric computations using Boolean operation within the Eclipse treatment planning system. The Dice similarity coefficient (DSC) can be used to quantify the performance of image segmentation techniques [11, 12]. DSC represent the size of overlap of two segmentations divided by the total size of both objects, and is expressed as:

$$ DSC=\frac{2\ \left|{V}_{GS}\bigcap {V}_x\right|}{\left|{V}_{GS}\right|+\left|{V}_x\right|} $$

where V_GS_ is the gold standard 10 phase PTV, and V_x_ is the compared PTV. A DSC score of 1.0 would mean the two volumes are identical, whereas 0.0 reflects no physical overlap. It is noted the PTV ratio analysis (Rx) does not take into account the physical location of the two volumes being compared for which in certain situations a large R value may have a corresponding small DSC score. To alleviate any confusion, from hence fourth we will reserve the terminology for validating contour overlap to be in “good agreement” when DSC > 0.700, as recommended by Zijdenbos et al. [13].

### Patient study

Seventeen patients who had previously undergone SBRT lung treatment were also included in this retrospective study. We primarily focused on lesions in the lower lungs where a range of movement up to 2 cm was recorded by analyzing tumor minima and maxima centroid-to-centroid displacements for all ten phases within the TPS by using the “move viewing planes to structure” feature. The overall tumor movement was accounted for in the medial-lateral (∆x), anterior-posterior (∆y), and superior-inferior (∆z) directions as given by the motion vector:

$$ M=\sqrt{\Delta  {x}^2+\Delta  {y}^2+\Delta  {z}^2} $$

Again, target volumes were segmented by one individual using the same lung window setting in all image sets.

## Results

### Phantom study

**Fig. 4:**
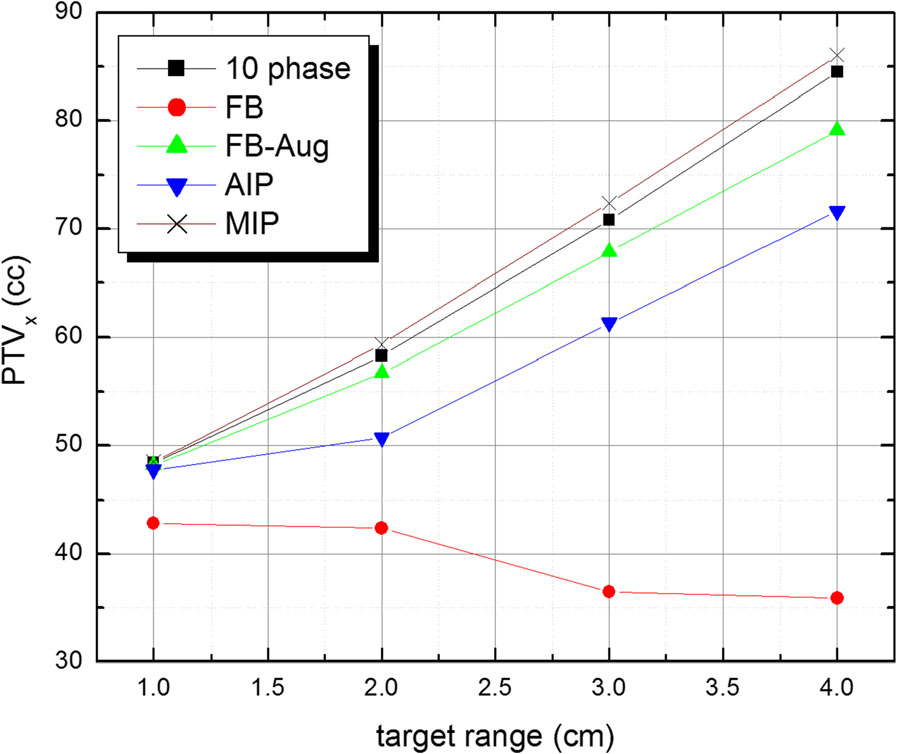
Phantom target volumes versus range of movement. PTV segmented from FB, FB-Aug, AIP and MIP 4DCT image sets are compared with respect to the 10 phase benchmark. A ± 0.5 cm tumor amplitude is represented by 1 cm target range. The negative slope associated with the FB method is attributed to the temporal resolution of the CT imaging system

Figure 4 shows the difference of PTVs generated by various techniques for target displacements ranging from ±0.5 to ±2.0 cm. With the exception of FB, Fb-Aug, AIP and MIP methods clearly show a linear increase in target volume for increasing range of motion along the superior-inferior direction. The negative slope associated with the FB approach (DSC = 0.740 - 0.408) is attributed to the interplay between CT data acquisition and target movement. Given our phantom study was performed at a fixed oscillation frequency of 15 cycles per minute for all ranges of motion, there exists a corresponding increase in target velocity as range increases. Considering the temporal resolution of the 4DCT imaging system is constant, the increased target velocity leads to residual motion artifacts which in this case results in a decrease in measured target volume. For all ranges of motion, a summary of the corresponding PTV ratios is given in Table 1, where subsequent DSC evaluations are indicated in parenthesis. For target displacement up to 1 cm, both FB-Aug (DSC = 0.913) and MIP (DSC = 0.913) methods have a PTV ratio of 98% with 10 phase gold standard technique, representing two viable time saving techniques for ITV delineation.

**Table 1:** Summary of experimental results. Ratio of Planned Treatment Volumes (PTVs) against 10-phase gold standard as a function of target motion. The Dice score for each scenario is indicated in parenthesis (DSC)

Range of Target	PTV_GS_	R_FB_	R_FB-Aug_	R_AIP_	R_MIP_
Motion (cm)	(cc)				
1	48.4	0.88 (0.740)	0.99 (0.913)	0.89 (0.886)	1.02 (0.913)
2	58.4	0.73 (0.633)	0.97 (0.905)	0.87 (0.860)	1.02 (0.910)
3	71.1	0.52 (0.515)	0.95 (0.884)	0.86 (0.864)	1.02 (0.894)
4	84.7	0.41 (0.408)	0.93 (0.877)	0.85 (0.850)	1.02 (0.879)

In theory, a maximal DSC = 1.0 would correspond to *R* = 1.00. In this case, both volumes are not only numerically the same but also occupy the same position in physical space. However, a perfect *R* value can have DSC = 0.0 which would indicate the two volumes are the same but have no spatial overlap. It is interesting to note in this phantom study the motion is restricted to only one dimension, for which the centroid positions are similar with the exception of the FB technique where there is a relative superior/inferior (Δz) shift with respect to other target delineation strategies. This is true because FB is only capturing a 3D snapshot of the target at a random location along the superior/inferior axis. Therefore, the fundamental difference between FB-Aug, AIP and MIP delineation strategies is the general shape when compared to ten phase. For *R* < 1 the physical volume was generally underestimated (ie; FB, AIP and FB-Aug), whereas when *R* > 1 the volume was overestimated (ie; MIP)

### Clinical study

**Fig. 5:**
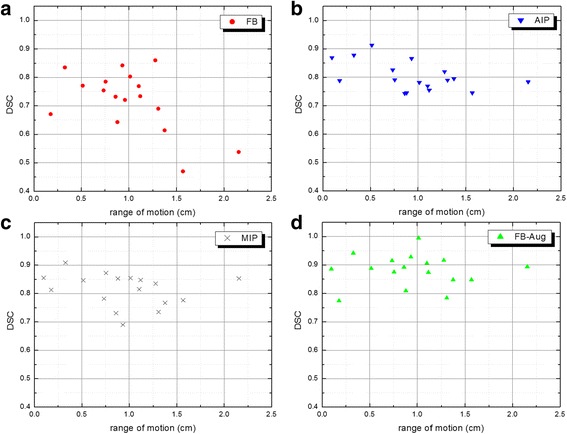
Clinical DSC scores as a function of tumor motion (M) displacements for (**a**) FB, (**b**) FB-Aug, (**c**) AIP and (**d**) MIP target delineations

**Fig. 6:**
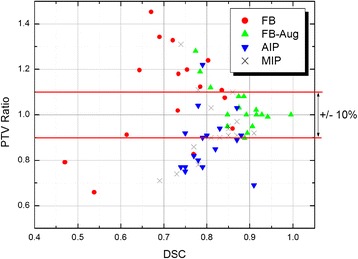
PTV ratio versus DSC score. Considering a PTV ratio acceptance window of ±10% with respect to the 10 phase benchmark, the FB-Aug scheme meets this criterion for 82% of clinical cases (DSC = 0.774-0.995), as compared to 65% for MIP (DSC = 0.690-0.909), and 41% for AIP (DSC = 0.743–0.913). The FB method faired the least at 34% (DSC = 0.470-0.860). As recommended in previous literature, “good overlap” occurs when DSC > 0.700 [13]

Patient data was collected from 17 cases with lung lesions displacements ranging from 0.1 to 2.2 cm. In comparison to all delineation strategies, the general inclination for FB method represented in Fig. 5 (a) shows the greatest decrease in DSC score for increasing range of motion. The association of the FB 10 mm fixed superior-inferior margin generally yields an overestimation of target overlap for small ranges of motion (< 1 cm), and an underestimation for larger motions, resulting in a mean DSC = 0.719 ± 0.106. Clinical results for the AIP technique are shown in Fig. 5 (b), where DSC = 0.803 ± 0.051. Consistent with our phantom study, DSC scores for the AIP method generally decreased with increasing range of motion, and were found to typically underestimate target coverage for which the mean PTV ratio was 0.88 ± 0.13, as illustrated in Fig. 6. MIP based delineation results are shown in Fig. 5 (c) where DSC = 0.816 ± 0.060. Contrary to our phantom outcomes, MIP more often than not underestimated target coverage, with an overall ratio of the PTVs of 0.95 ± 0.15. Finally, when compared to single CT image target delineation, the FB-Aug approach as shown in Fig. 5 (d) nominally exhibited superior target overlap for all ranges of motion yielding a mean DSC = 0.881 ± 0.056. A summary of patients results are listed in Table 2.

**Table 2:** Summary of clinical results. Ratio of Planned Treatment Volumes (PTVs) against 10-phase gold standard as a function of target motion. The Dice score for each scenario is indicated in parenthesis (DSC)

Patient	Target Range	PTV_GS_	R_FB_	R_FB-Aug_	R_AIP_	R_MIP_
No.	M (cm)	(cc)				
1	1.3	223.9	0.94 (0.860)	1.00 (0.916)	0.85 (0.820)	0.90 (0.835)
2	0.3	24.5	1.11 (0.835)	0.99 (0.942)	0.91 (0.878)	0.92 (0.909)
3	1.1	124.2	0.83 (0.769)	0.95 (0.906)	0.82 (0.769)	0.90 (0.815)
4	0.7	27.6	1.20 (0.754)	1.02 (0.915)	0.94 (0.826)	1.11 (0.782)
5	1.6	24.8	0.79 (0.470)	1.00 (0.848)	0.75 (0.745)	0.78 (0.776)
6	1.4	23.0	0.91 (0.614)	0.95 (0.848)	0.91 (0.795)	0.86 (0.768)
7	0.8	61.8	1.12 (0.785)	1.03 (0.874)	0.90 (0.791)	0.97 (0.872)
8	0.9	21.4	1.19 (0.643)	1.12 (0.809)	0.77 (0.745)	0.94 (0.853)
9	2.2	63.4	0.66 (0.538)	0.92 (0.894)	0.80 (0.784)	0.95 (0.853)
10	0.9	109.6	1.07 (0.842)	1.00 (0.928)	0.89 (0.866)	0.91 (0.872)
11	0.5	23.1	0.82 (0.770)	0.90 (0.888)	0.69 (0.913)	0.71 (0.690)
12	1.1	51.5	1.18 (0.734)	1.08 (0.874)	0.92 (0.755)	0.91 (0.848)
13	1.3	29.8	1.34 (0.690)	1.19 (0.784)	0.77 (0.790)	1.10 (0.861)
14	0.2	52.6	1.45 (0.671)	1.28 (0.774)	1.22 (0.788)	1.31 (0.735)
15	1.0	40.1	1.24 (0.803)	1.00 (0.995)	1.04 (0.783)	1.03 (0.813)
16	0.1	18.3	1.33 (0.721)	1.08 (0.886)	1.03 (0.869)	1.10 (0.855)
17	0.9	33.4	1.02 (0.732)	1.00 (0.892)	0.77 (0.743)	0.74 (0.731)

## Discussion

Although previous studies have looked into delineation strategies based on PTVs derived from alternative 4DCT datasets, to the best of our knowledge this is first study to examine target conformance as function of range of movement. In both experimental and clinical scenarios, PTV’s derived from the FB image set are the least equivalent with the 10 phase benchmark for all ranges of motion. The declination of FB target coverage for increasing range of motion is attributed to standard 10 mm extension of the superior-inferior PTV margin which generally leads to an overestimation of target coverage for lesser ranges of motion, versus underestimated coverage for large tumor displacements.

In general, the AIP scheme both clinically and experimentally favors a decrease in target coverage with increasing range of target movement. Experimentally, the decline in AIP volumetric representation for target movement exceeding 1 cm is attributed to peripheral lower density pixel averages due to increased target velocity, as such is case with the FB scenario. Clinically, it is clear AIP delineation is deviating from the standard bench mark as lesser target volume is taken into consideration for increasing range of motion.

In our phantom study we found MIP volumetric segmentation consistently exceeded the 10 phase standard for all ranges of motion by approximately 2% (DSC > 0.878). The underlying principle of MIP image generation involves assigning the highest density from all 10 individual phases at any point in 3D space. Theoretically a MIP image should give the maximum tumor delineation, which is consistent with our experimental results, keeping in mind the phantom target was entirely surrounded by low density media while undergoing simple harmonic motion. However, in clinical cases MIP more often than not underestimated target coverage resulting in an overall ratio of the PTVs of 0.95 ± 0.15. It is noted when the breathing is irregular, or similar density as that of tumor is nearby, MIP results can significantly differ from the 10 phase standard [14]. The former is attributed to keeping only one CT slice per table position for any phase resulting in discarding a large number of CT slices containing relevant data due to irregular breathing patterns. Zamora et al. have accordingly recommended that all 4DCT images, before the phase binning itself, should be used for MIP generation to ensure the image set captures the entire excursion of the tumor movement which may even yield a smaller volume than the 10-phase gold standard [6]. However, inclusion of all 4DCT images increases the noise in the image and may blur the tumor edges making it difficult for segmentation particularly if any structure of similar density is proximate [15]. Accordingly, Muirhead et al. have suggested MIP usage only for stage I lung cancers [16].

In both clinical and experimental scenarios, the FB-Aug approach was found to be in good agreement with respect to 10 phase benchmark. Comparatively, the accuracy of the FB-Aug technique takes into consideration the tumor positions at extreme minima and maxima locations while reducing the potentiality of a geographic miss. We found the conformance using the FB-Aug approach to slightly underestimated target coverage in our phantom study, whereas for clinical cases in general an overestimation in coverage was observed for which the PTV ratio was 1.03 ± 0.09. Nonetheless, it is important to note target coverage remained reasonably consistent regardless of range of motion. Therefore, when considering delineation efficiency relative to the 10 phase benchmark, the FB-Aug technique may be considered a potentially proficient and viable clinical alternative, particularly when 4DCT imaging is not available.

Certain limitations of the phantom study need to be addressed when compared to clinical cases. First, the period and amplitude of the tumor movement in the phantom was consistent. Second, within the phantom the tumor was only moving along one axis. Finally, the phantom tumor is also rigid and non-deformable. In consideration to real patient scenarios, irregular breathing patterns, 3D motion, and tumor’s deformation gives rise to additional errors which experimentally represent the upper bounds of what may be considered the best case scenario. Although the use of 4DCT allows for more accurate target volume segmentation by accounting for respiratory motion, further assessments into target range, rate of mobility and trajectory need to be made in order to efficiently generate a suitable PTV for lung lesions which we intend to address in our future study.

## Conclusions

A comparative analysis for various target volume delineation techniques is presented. Our results indicate the range of motion dictates the accuracy of the PTV defined by FB, FB-Aug, AIP and MIP image segmentation. In clinical scenarios for lung tumor displacements up to 2 cm, we found best conformance using the FB-Aug technique comparable to the 10 phase benchmark, followed by the MIP approach, then AIP, and finally FB. For PTV structures based on the delineation of the single phase FB image set, we have shown even with 10 mm superior-inferior margin [5 mm axial] yields suboptimal tumor coverage potentially leading to errors in dose delivery. Among the various techniques used for PTV generation for a moving target, a judicious balance between accuracy and work-flow efficiency is required to ensure expected clinical results.

## Abbreviations

4DCT: Four Dimensional Computed Tomography
**AIP**: Average Intensity Projection,
**DSC**: Dice Similarity Coefficient
**FB**: Free Breathing
**FB-Aug**: Free Breathing Augmented
**GTV**: Gross Tumor Volume
**ITV**: Internal Target Volume
**MIP**: Maximum Intensity Projection
**PTV**: Planned Target Volume
**SBRT**: Stereotactic Body Radiation Therapy
**TPS**: Treatment Planning System
